# The role of inflammation in the immune evasion of KRas

**DOI:** 10.3389/fimmu.2026.1831303

**Published:** 2026-05-07

**Authors:** E. Jane Homan, Robert D. Bremel

**Affiliations:** ioGenetics LLC, Madison, WI, United States

**Keywords:** caspase, cathepsin, epitope, immune evasion, immunotherapy, inflammation, KRas, trypsin

## Abstract

KRas, NRas and HRas mutations are recognized in over 25% of all tumors, with the predominant mutations occurring at amino acids G12 or G13. While small molecule inhibitors of KRas show therapeutic promise, KRas has largely resisted control by immunotherapy in clinical cases, although immune responses may be detected following vaccination. Inflammation is a recognized precursor of most KRas-associated tumors. In inflammation cathepsin B leaks from the lysosome and at the higher pH of the cytoplasm acquires endopeptidase activity, in addition to its exopeptidase role. Cathepsin B is consistently upregulated in tumors and its role in tumorigenesis has been attributed to increased apoptosis and digestion of the extracellular matrix. Here we examine the effect of cathepsin B on neoepitopes in KRas. We predict that cathepsin B cleavage patterns of KRas may lead to the destruction of the G12 and G13 mutant neoepitope peptides that would otherwise bind to MHC I, thereby rendering them immunologically invisible. We review reports of the interaction of cathepsin B with trypsinogen in the pancreas and caspases in inflammasomes and the potential effect of premature activation of trypsin on immune evasion of G12R mutants. We summarize our observations and literature review in a schematic describing the potential role inflammation and the actions of cathepsin B, trypsin, and caspases on the immune evasion of KRas and related Ras family gene products.

## 1 Introduction

Mutants of KRas, and the closely related HRas and NRas genes, are recognized as oncogenes in 25-30% of cancers, including 90% of pancreatic adenocarcinomas (PDAC), and a large proportion of non-small cell lung cancers (NSCLC), colorectal cancers (CRC), and hematologic cancers (1–5). The structure and function of the Ras genes has been intensively studied since their discovery over 40 years ago (6, 7). Until recently KRas has been considered “undruggable”. This has been partly addressed by the recent development of small molecule inhibitors, first for G12C KRas mutants (8), and more recently for G12D and G12V mutants (9, 10), although with limited duration of efficacy (11). Yet the means by which mutants of KRas, NRas and HRas evade immune control remains an enigma.

Many of the studies of KRas have been conducted in cell lines and mouse models which lack the complexity of a naturally developing tumor, and the observations made in such models fail to replicate *in vivo* (12, 13). Immunoinformatics can provide the opportunity to analyze complex multivariate interactions which may be overlooked in model systems. By combining our own immunoinformatics analyses with a review of reported experimental, clinical and contextual information developed by others, we propose a hypothesis that may explain the immune evasion and the difficulty of immunotherapeutic targeting of KRas in tumors. We examine the apparent impact on KRas of enzymes that are upregulated in inflammation and conclude that they may play a key role in degradation of KRas neoepitopes, rendering these immunologically invisible. We acknowledge that the literature cited here is but a limited subset of the over 30,000 peer reviewed publications on KRas and related Ras oncogenes.

If further validated, this hypothesis raises additional challenges and opportunities for intervention and opens up questions regarding the structural stability of the Ras oncogenes in inflammation. We hope this discussion will contribute to debate around a new paradigm for understanding KRas.

## 2 Brief background

The Ras superfamily of proteins comprise small guanosine triphosphatases (GTPases), that hydrolyze GTP to GDP, and which are highly evolutionarily conserved. In humans there are over 150 members of the family, each playing a different role in regulating the balance GDP and GTP binding and sharing the common sequence motifs GXXXXGKS/T (14–16). KRas, HRas, and NRas are identical in their first 86 amino acids. Mutants of each of these differ in their tissue distribution and pathogenesis (17). For convenience we will focus here largely on KRas, however it will become evident that much of the discussion that follows can be applied to the common mutations of NRas and HRas.

KRas, when bound to GTP, activates several downstream effector pathways, principally the MAPK, mTOR and RalGEF pathways, which collectively control cell growth, replication and survival (18, 19). While individual KRas molecules are often described as a “switch” controlling the binding of GDP and GTP and engagement and activation of downstream pathways, it should be understood that at a whole cell level the aggregate status of multiple KRas molecules behaves more like a rheostat. The engagement of the downstream pathways acts through binding to two sites: the so-called switch-I (amino acids 30-40) and switch-II (amino acids 60-76) (20, 21).

The vast majority of KRas mutations occur at positions G12 or G13 located in the P loop, which links the beta-1 strand to the first alpha helix of the molecule, and lies on the N terminal side of the GDP-GTP binding site (1, 20, 22). For an example structural model see https://www.rcsb.org/structure/6GOD (23). When KRas is mutated at positions G12 or G13 GTPases are sterically blocked from hydrolyzing GTP, so the mutated KRas remains in an activated state for longer, promoting persistent cell replication and tumor development (24). Mutation of KRas is not sufficient alone to trigger tumorigenesis. Many non-tumor cells carry KRas mutations (7, 25, 26). Genomic mapping studies of normal pancreatic tissue have shown that multiple different mutations of KRas may be present without any tumor progression and that when tumors arise, it is from clonal expansion of a cell bearing a single mutant from among the several different mutants present (27). Some other triggering event is needed for transformation in cell lines, and additional mutations are typically found at the time tumor cases present clinically (28, 29). The triggering events in cell lines may differ from those in a natural tumor setting and tumor development may proceed for an extended time before clinical signs are manifested.

## 3 Immune evasion and KRas

**Figure 1:**
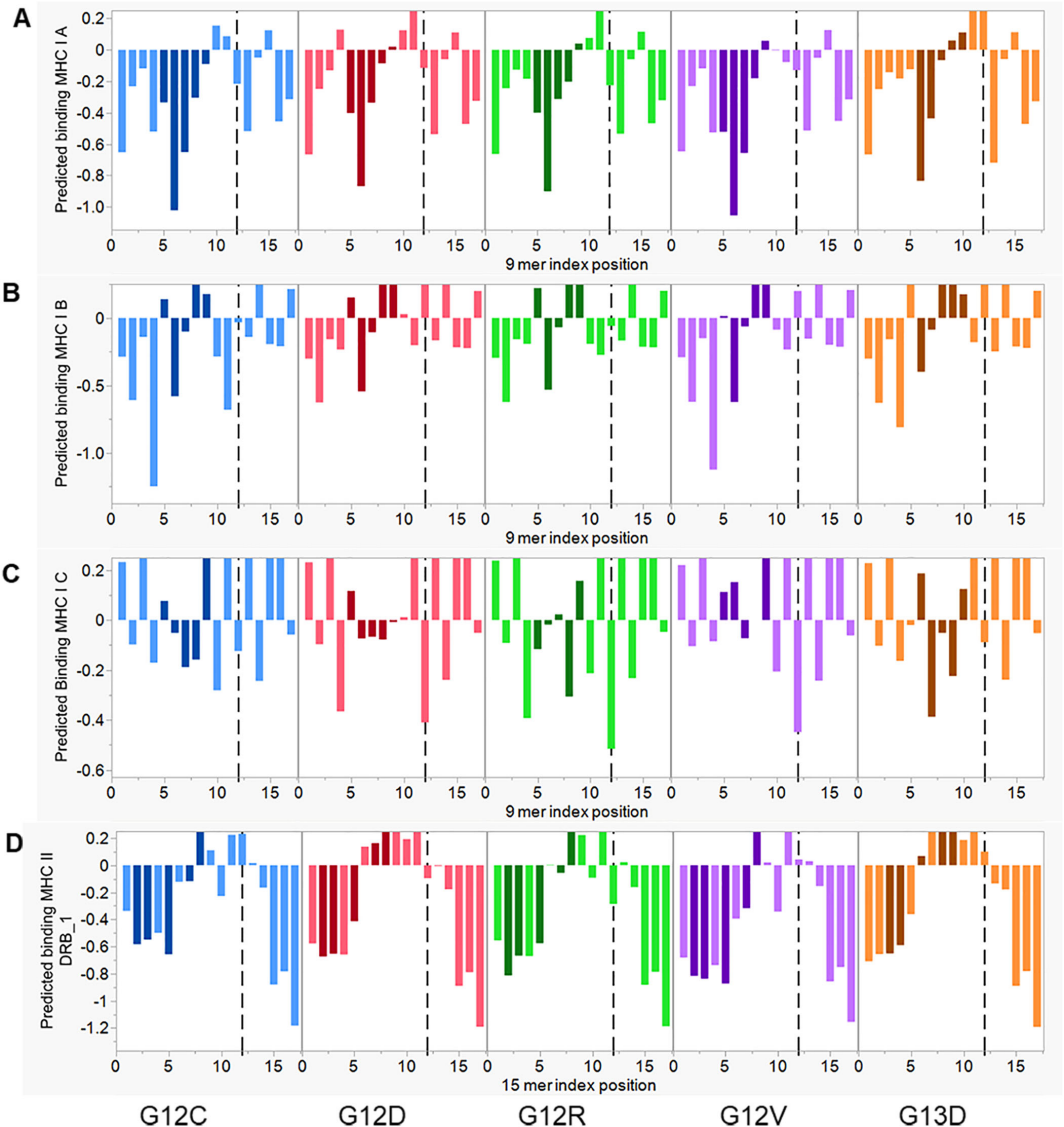
Predicted MHC binding of the common KRas mutations. Predicted mean Z scaled MHC binding for the peptides spanning the common mutant positions in KRas, shown in average standard deviation units relative to all peptides in KRas, where highest binding affinity are the lower values. The X axis shows the index position of each sequential peptide. Dotted line indicates position of amino acid 12, or mutant thereof. Shaded positions are those in which the mutant would be exposed to a TCR. **(A)** Mean of 31 MHC I A alleles; **(B)** Mean of 31 MHC I B alleles; **(C)** Mean of 8 MHC I C alleles; **(D)** Mean of 17 MHC II DRB1 alleles.

The molecular landscape in tumors exhibits multiple mechanisms of immune escape of mutations, precluding neoepitope peptide presentation and T cell engagement. The role of the tumor microenvironment has been widely discussed (30). At a molecular level we have described escape of the expressed mutants of other major oncogenes and tumor suppressors as a function of three factors: the absence of MHC binding of a mutant-bearing peptide precluding presentation of a peptide-MHC (pMHC) to a T cell receptor (TCR); a dominant pMHC binding register that places the mutant amino acid in a position hidden from the TCR (typically position 2 or 9 of a 9mer peptide corresponding to the anchor positions of an MHC I); and thirdly, the mutant creating rare T cell exposed motifs that elicit few responding cognate T cells (31). A combination of these evasion mechanisms provides an explanation for the immune escape of mutants of other major gene products whose mutants contribute to the top 100 tumor mutations and most cancer cases. However, KRas peptides that span positions G12 and G13 are predicted to have adequate MHC I and MHC II binding for many common HLA alleles, allowing them to be presented to T cells (**Figure 1**). HLA binding of multiple mutant KRas neoepitopes have also been demonstrated in cell culture (32). Peptides comprising the G12 and G13 mutants have T cell exposed pentamer motifs that are highly represented in the human proteome and the gastrointestinal microbiome, indicating that availability of cognate T cells should not be a constraint to T cell responses (31). Only G12D creates one T cell exposed motif which is not represented in the human proteome. Multiple changes in the tumor microenvironment come into play in KRas, as in all tumors (33), however it is evident that KRas, NRas and HRas stand apart from other major oncogenes and must have a different route of immune evasion.

Vaccines directed to KRas have not been broadly successful in containing progression of tumors comprising KRas mutants. Vaccination with peptides, or peptides expressed from mRNA, have been shown to generate immune responses in mice (34–36) and in patients with KRas mutant tumors, in some cases when combined with checkpoint inhibitors (37–42). Vaccines which comprise long sequences do not always differentiate responses to mutated neoepitopes from unmutated epitopes. Some extended progression-free survival has been reported in PDAC vaccine applications, however KRas mutants have been combined with other neoepitopes which may have contributed to the outcome in the absence of responses detected to KRas peptides (40, 42, 43).

Similarly, checkpoint inhibitors alone have not shown significant success in curtailing progression of KRas tumors (43–46). In CRC it is noted that microsatellite instability (MSI) favors a response to checkpoint inhibitors, whereas microsatellite stable CRC, comprising KRas mutants, are not responsive to checkpoint inhibitors. Likewise, the relatively few PDACs with MSI are more responsive to checkpoint inhibitors than the majority that carry KRas mutations (47, 48).

The successful application of T cell adoptive transfer targeting a KRas G12D mutant was reported in 2016 (49). This involved T cells targeting a peptide presented by a C*0802 MHC allele. In this case the target peptide (GADGVGKSA) places the mutant aspartic acid residue in an anchor position (position 3 of a 9mer), where it is not engaged by a T cell receptor and the N terminal of the peptide is at position 10. Consequently, it appears the T cell engagement is facilitated in this case by a minor difference in MHC binding affinity in the mutant compared to the wildtype. In the decade since, reports of further success with adoptive T cell transfer of KRas responsive T cells have been elusive. While immunopeptidomic detection of intact mutant KRas peptides is demonstrable in cell lines (32, 50), reports of detection in tumor tissue are lacking (51–53). Others have puzzled at the lack of KRas responsive T cells in clinical cases (5, 54).

## 4 Predicted cathepsin B cleavage

In assembling a comprehensive immunoinformatics analytical platform, as a function of developing predictors of MHC binding, we also built predictors of cathepsin L and S cleavage which are routinely included in our workflow to evaluate T cell epitopes in any protein. As training sets were available, we also included prediction of cleavage by cathepsin B (55). More recently additional reports of cathepsin function have become available (56) and we have updated and refined the predictors. The application of this cathepsin cleavage prediction has been described previously (57, 58).

Briefly, the system comprises a contrastive learning approach using the physical properties (59, 60) of the amino acid octamer surrounding each of the 400 possible P1P1’scissile bonds, using the convention for peptidase specificity description to train neural networks (61). For the contrastive training, the octamer of a scissile bond dimer that is cleaved is paired with another octamer with the same dimer in the same protein that was not cleaved (62). This process is repeated by bootstrap aggregation (bagging) to create multiple random paired ensembles of predictors for as many P1P1’ that are cleaved in the mass spectrometry peptide fragment datasets used for training. The learning process creates aggregate values and AUROC values for both cleavage and non-cleavage for all scissile bond pair found in the training sets. The overall average AUROC at pH 6 for cathepsin B cleavage was 0.84 ± 0.04 and for non-cleavage is 0.74 ± 0.07, but for the more common scissile bonds such as those in KRas noted below the values extended to 0.91. The process is repeated for each pH independently and an ensemble mean and standard deviation cleavage prediction is made for each potential scissile bond in a protein sequence. We have found a cleavage probability of 0.8 to be a practical threshold to use in experimental work (58). Supplemental **Supplementary Figure S1** shows predicted mean and standard deviation at every KRas P1P1’ position.

**Figure 2:**
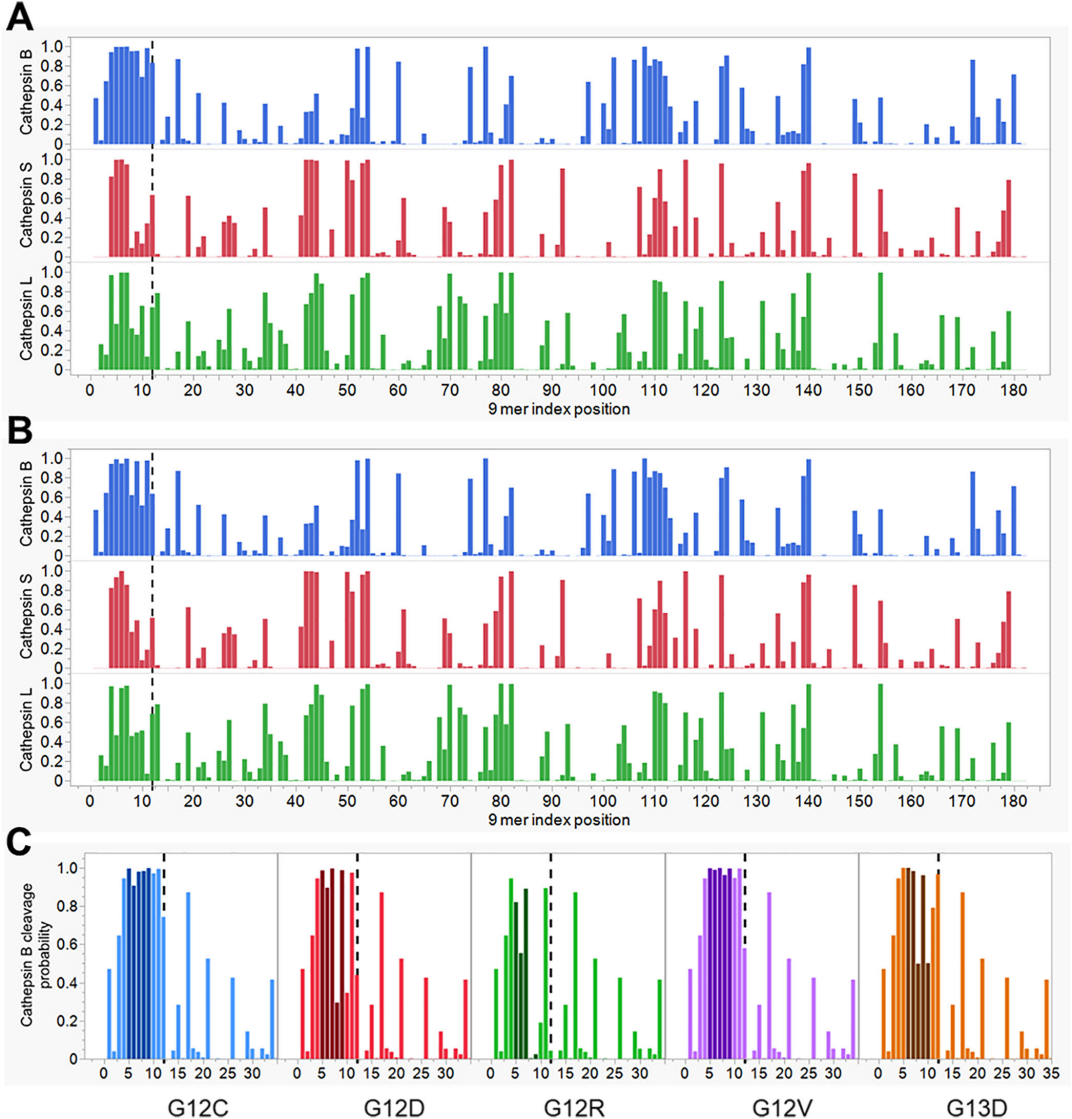
Predicted cathepsin cleavage of KRas. The X axis shows the index position of each sequential 9 mer peptide from N to C terminal of KRas. The dotted line indicates amino acid position 12. The Y axis shows the predicted probability of cleavage of the peptide at the P1P1’ dimer comprising amino acids 4 and 5 or that 9 mer by the cathepsin indicated. Based on experimental data and AUC in neural net training predictions with a predicted probability of 0.8 is considered positive for a high probability cleavage event. **(A)** Wildtype KRas, showing all positions in the protein and predicted cleavage by cathepsin B (top tier), cathepsin S (middle) and cathepsin L (bottom tier). **(B)** As for A but showing predicted cleavage of G12D mutant. **(C)** Predicted cathepsin B cleavage in the initial 35 amino acids of the common mutants of KRas, G12C, G12D, G12R, G12V and G13D shaded bars are those peptides wherein the mutant would be exposed to a TCR.

Analysis of predicted cathepsin B cleavage of KRas identified an unusual pattern. As shown in **Figure 2** each of the G12 and G13 mutants and the wildtype KRas show a series of high probability cleavage sites impacting peptides which comprise the commonly mutated amino acids 12 and 13. If the protein is cleaved at the high probability sites shown in **Figure 2**, those peptides that would otherwise present the mutant neoepitopes are destroyed and the mutants become immunologically invisible.

**Figure 3:**
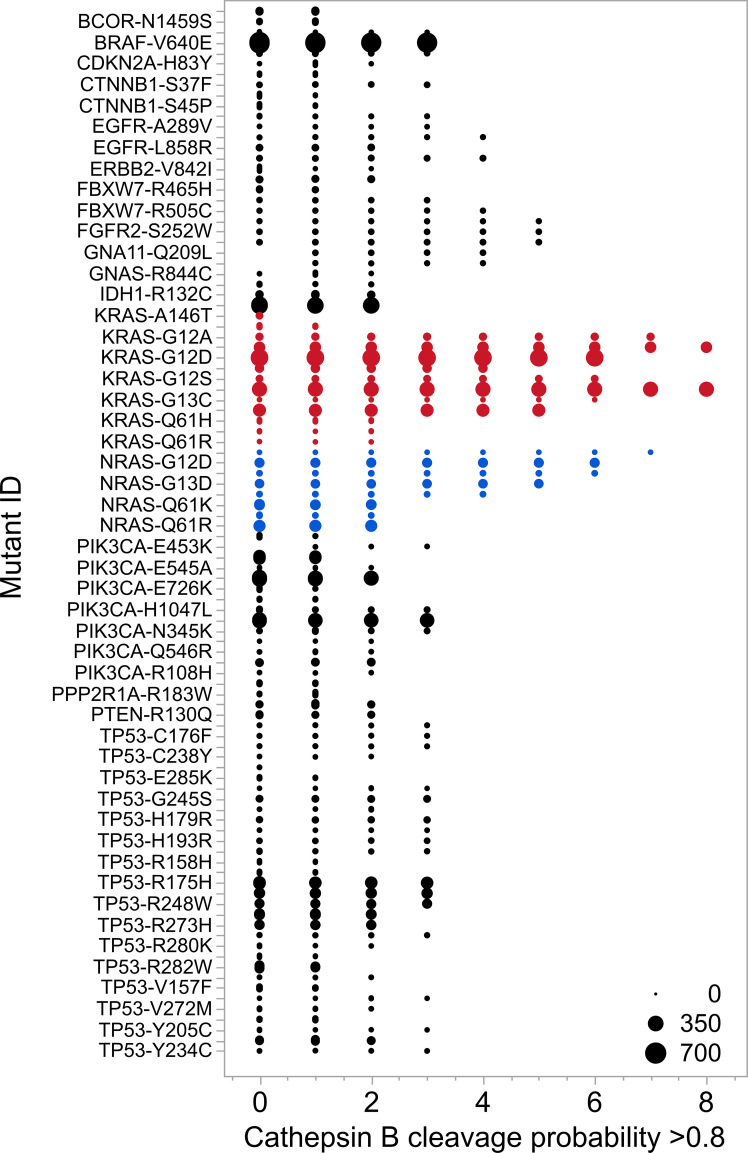
Comparative cathepsin B cleavage probabilities for the 100 most common mutants found in all tumors. The Y axis lists the most common tumor mutations listed in the Genome Data Commons (145) in May 2025. X axis shows the number of dimers within the 9 mer that comprises the mutant amino acid for which the probability of predicted cathepsin B cleavage exceeds 0.8. Case numbers are reflected in the size of the markers. KRas and NRas mutants are shown in red and blue respectively.

When compared with predicted cleavage patterns in peptides that comprise the high frequency mutations in other common oncogene and suppressor gene products, the Ras G12 and G13 mutations are clearly different (**Figure 3**). In most unrelated proteins high probability cathepsin B cleavage predictions are seen only at single dimer sites scattered along a protein. A contiguous series of multiple high probability cathepsin B cleavage in KRas is highly unusual. **Supplementary Figure S2** shows the cleavage pattern in wildtype TP53 for comparison.

## 5 Cathepsin B in tumors

Cathepsin B is unique among the cathepsins and cysteine proteases in that it is widely distributed in most cell types (63). In normal cells cathepsin B is located in lysosomes, where at low pH (pH4) it functions as an exopeptidase (a dipeptidyl carboxypeptidase). At higher pH (pH6-7), to which it is exposed upon leakage from lysosomes into the cytoplasm, cathepsin B acquires endopeptidase activity, up to a pH of 7.2 at which it is reportedly inactive (64–66). The alternate pH dependent function is triggered by the release of salt bridges which hold a 20 amino acid loop (“the occluding loop”) in place over the endopeptidase binding site at low pH but expose it at higher pH (65, 67–69). Cathepsin B differs from cathepsins L and S in the presence of the extended occluding loop. In inflammation, lysosomes leak cathepsin B into the cytoplasm and this contributes to further activation of the inflammasome (70–75). Cathepsin B released from lysosomes locates adjacent to the cell membrane, effectively co-locating with KRas (76–81). *In vitro* the simultaneous action of endo- and exopeptidase functions at cytoplasmic pH produces ragged cleavages with several adjacent cleavages rather than discreet cleavage events. This is a characteristic that is not seen in standard mass spectrometry proteomics using trypsin fragmentation and complicates the proteomic analysis of cathepsin B (unpublished data).

Cathepsin B is consistently upregulated in tumors (82, 83), in some cases becoming one of the most clearly upregulated genes and considered a biomarker for poor prognosis (82, 84–86). In mouse models the expression of cathepsin B increases with tumor progression (87), but administration of cathepsin B inhibitors and cathepsin B gene knockout slow progression of pancreatic tumors in mouse models (88).

The adverse effect of upregulation of cathepsin B on tumor progression has been logically attributed to its effect on the extracellular cell matrix, facilitating metastasis, and to an increase in apoptosis and autophagy (89–93). What has not been addressed, in addition to these effects, is the potential impact of cathepsin B cleavage on epitope presentation and immune evasion.

## 6 Inflammation

Inflammation has been called a “hallmark” of cancer (94, 95). Inflammation can occur as a consequence of tumor progression and therapy (96). But inflammation is also a critical precursor to cancer, and this is true of tumors which have a high prevalence of KRas mutations. Pancreatitis is a recognized precursor of PDAC (97–99). Particulate air pollution, smoking or second-hand smoke lead to inflammation and are antecedents of lung cancer in smokers and non-smokers (100–103). Inflammation and dietary changes are thought to contribute to the rising numbers of CRC. Inflammatory bowel disease has also been noted as a risk factor for CRC (96, 104, 105). *Heliobacter pylori* is a risk factor associated with both chronic inflammation and gastric cancer with KRas drivers (106, 107). Chronic inflammation is a noted risk factor in cholangiocarcinoma (108). More generally, inflammaging has been considered a driver of cancer incidence in the elderly (109).

In the present context, whether a transient or dynamic inflammatory event results in lysosome leakage and cathepsin B endopeptidase activity, or whether a chronic inflammatory state is required is unknown. Inflammation may provide the pivot point between equilibrium with a tumor and its immune escape. Lysosomal integrity is not “all or nothing” and there is increasing evidence that some degree of lysosomal permeability is commensurate with healthy cell function (110–112).

## 7 Other enzymes

Cathepsin B functions in the context of other peptidases, including with trypsin in the pancreas and caspases in inflammasomes. We therefore considered the possibility of synergy with these proteases.

The early intracellular activation of trypsinogen to trypsin is a driver of pancreatitis (97). While premature trypsin activation is a source of inflammation, which may in turn drive cathepsin B release, it has also been shown that cathepsin B can contribute to trypsin activation (113, 114). Notably, trypsin once activated cleaves proteins on the C terminal side of an arginine residue (61). The detection of KRas G12R mutations is disproportionately higher in PDAC relative to other KRas tumors (1, 115). To our knowledge, the effect of trypsin on KRas has not been reported. Our predictions of cathepsin B cleavage of KRas (**Figure 2**) show a lower probability of cleavage of G12R compared to G12D and G12V. However, the combination of cathepsin B and trypsin cleavage of the G12R neoepitopes may contribute to the higher prevalence of G12R mutations in PDAC.

Caspases are the primary enzymatic effectors in inflammasomes (116); in humans the inflammatory caspases comprise caspase 1, 4, 5 and 12. The preferred site of cleavage of all the caspases is on the C terminal side of an aspartate residue, and yet further preferentially at an aspartate-glycine (DG) dimer (61). This potentially favors immune escape through cleavage by caspase of a KRas G12D mutant which creates a *de novo* DG dimer (which is not the case in a G13D mutant), complementing any cathepsin B activity. A further DG dimer is present at amino acids 47–48 in the inter-switch region of KRas.

Within the pancreas caspase may also act on trypsinogen at the DDDD motif adjacent to the enterokinase cleavage site. Furthermore, a hereditary mutant of trypsinogen comprising D22G which creates a DG dimer caspase cleavage site, is a known risk factor for pancreatitis (117).

## 8 Connecting the dots

**Figure 4:**
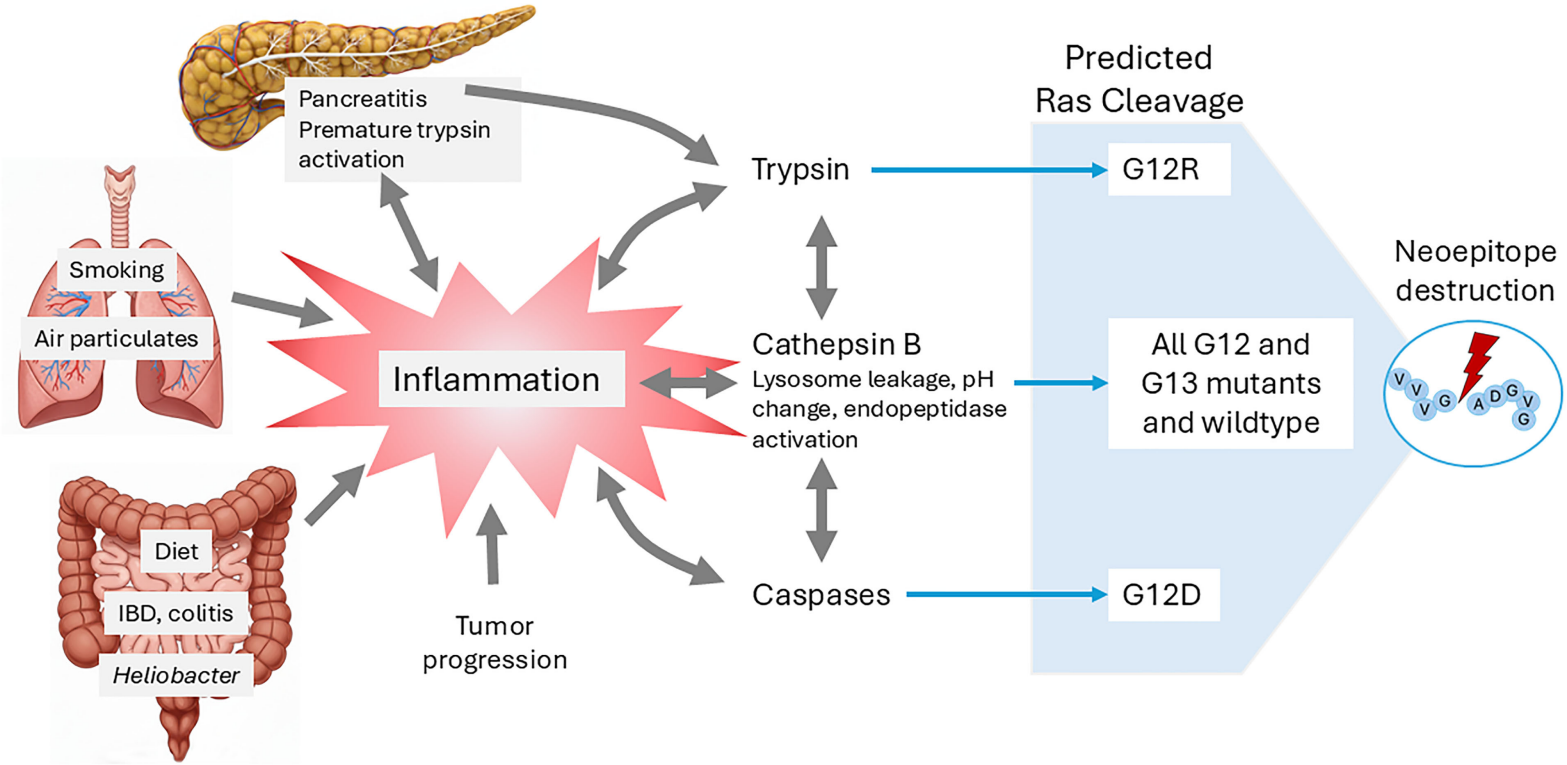
Schematic of the relationship between inflammation, enzyme cleavage and KRas neoepitope loss.

The schematic in **Figure 4** combines the interactions we have documented. The supporting literature is shown in **Table 1**. This documents many interactions in which it is not clear which is cause and which is the result. But most of the molecular interactions depicted are likely a component of a feed-forward loop, where once a synergistic process of inflammation and protein cleavage has started, it continues to progress. The central thread is that inflammation and epitope cleavage may lead to immune invisibility of the common KRas G12 and G13 mutants. It is likely that, in an inflamed tumor cell, a combination of different states co-exist with KRas activated and not, cleaved and un-cleaved. The balance of these may also change over time and the combination differ in different tissues.

**Table 1:** Summary of information contributing to hypothesis.

Protease	Feature cited	Citation or prediction
Cathepsin B		
	Cathepsin B in all cell types	(63)
	pH4 exopeptidase- pH6–7 endopeptidase activity	(64–66)
	Lysosome leakage in inflammation	(65, 67–69, 116)
	Cathepsin B involved in inflammasome activation	(70–74)
	After lysosome leakage cathepsin B location is perimembraneous	(76–81)
	Upregulated in tumors, increases with progression	(82, 83, 87, 132)
	Cathepsin B is a biomarker of poor prognosis	(82, 84–86)
	Role in tumor invasiveness, autophagy	(89–93)
	Cathepsin B inhibitors slow progression of PDAC in mice	(88)
	Cathepsin B cleavage of KRas wild type and mutants in P loop	*In silico* prediction
Trypsin		
	Premature activation of trypsinogen leads to pancreatitis	(97, 98, 133, 134)
	Pancreatitis is a precursor to PDAC, human and mouse models	(99, 135)
	Cathepsin B exacerbates trypsinogen activation -	(113, 114, 136)
	Cathepsin B inhibition reduces trypsinogen activation	(137)
	G12R is higher frequency in PDAC than other KRas related tumors	(1, 115)
	Trypsin cleaves on the C side of arginine	(61)
	Trypsinogen mutation D22G that creates a DG dimer is a risk factor for PDAC	(117)
Caspase		
	Caspases 1,4,5,12 are active in inflammasomes	(116)
	Caspases may exacerbate inflammation	(138)
	Caspases are upregulated in tumors	(139–141)
	G12D activates inflammasomes and caspases in myeloid malignancies	(142, 143)
	Preferred cleavage site of caspases is a DG dimer.	(61)
	G12D is the most common KRas mutant in tumors	(1)
	A G12D mutation in Kras creates a *de novo* DG dimer	(18, 20)
	A DG dimer is present at positions 47–48 of Kras between the switch sites	(18, 20)
	Trypsinogen mutation D22G creating a DG dimer is a risk factor for PDAC	(117)
	Trypsinogen DDDD motif adjacent to the enterokinase cleavage site	(144)

## 9 Implications for intervention

In the absence of any inflammation-related cleavage the intact peptides spanning KRas G12 and G13 mutations show characteristics of cathepsin L or S cleavage, MHC binding and T cell exposed motif frequency that indicates that they should be recognized as neoepitopes in most patients, engaged by T cells and eliminated. This is supported by the demonstration of detectable peripheral T cell responses in mice and humans following vaccination and in clinical disease (34, 41, 118, 119).

If cleavage can be inhibited in the tumor, then the recognition of KRas mutations should, in principle, be restored. This would open the way to effective therapeutic vaccination and checkpoint inhibitor therapy. Loss of integrity of the KRas molecule may also be a contributing factor in development of resistance to the G12C inhibitor drugs (11); inhibition of cleavage may therefore extend efficacy.

Not all molecules of KRas in a cell are likely affected in unison. It is the aggregate balance of peptide cleavage and epitope presentation, in combination with engagement of downstream pathway effectors that may affect outcome. To the degree that more intact epitopes may be presented to effector T cell engagement, the more likely the mutant-bearing cell would be subject to effective immune control.

Several cathepsin B inhibitors have been used in settings other than KRas tumors (120–123), and have been shown to slow other tumor progression in mice (124). However, they have not been evaluated in KRas tumors and there is a need to carefully balance specificity and cell penetration (122) of each potential inhibitor.

## 10 Possible wider implications and open questions

At cytoplasmic pH cathepsin B functions as both an endopeptidase and an exopeptidase. Not only does this complicate the detection and identification of cleaved fragments, but it also means that consideration should be given to the possibility of dipeptidyl carboxypeptidase action on the C terminal of the KRas molecule, affecting its prenylation and membrane attachment (125, 126).

Beyond the possibility of immune invisibility, there is the question of what happens to molecular stability if the P loop of KRas is cleaved by cathepsin B, given the positioning of the initial ~12 amino acid beta-1 strand as a central core of the molecule (20). Also, cleavage at the canonical caspase site between the switch I and switch II domains would split the molecule resulting in loss of allosteric control of the two switch regions. A combination of these molecular events might occur. While complete disorganization is a likely outcome, it is also feasible that binding of the switch sites to downstream pathways could be enhanced by the predicted P loop cleavage and altered molecular structure. Studies of KRas engagement of effector ligands have almost exclusively assumed intact molecules. In one exception it was shown that removal of just the N-terminal methionine changed the structure of KRas in the switch region (127).

The exact role of wild type KRas relative to mutants in early tumorigenesis *in vivo* is not fully understood. This opens the question of what is the effect of inflammation and cleavage on wild type KRas. Unmutated KRas shows similar P loop cathepsin B cleavage probability as the mutants (**Figure 2**). It has been reported that wildtype counterparts of the Ras oncogenes have a tumor suppressor function which, if lost, can facilitate tumor progression (128). A residual level of wildtype KRas functionality may be essential to downstream signaling to avoid apoptosis (129). Another member of the Ras family, RRas2 without mutations has been shown to be upregulated and to drive chronic lymphocytic leukemia (130) and has been linked to the progression of triple negative breast cancer (131). The detection of mutants of HRas in cell cultures was fundamental to the initial recognition of Ras oncogenes (7) and mutations of KRas and related genes have been followed as biomarkers ever since. But cell cultures do not experience inflammation and continuous cell cultures have other oncogenes. If wild type KRas undergoes the same cleavage as the G12 and G13 mutants, does this imply that wild type KRas can serve as a tumor initiator in inflammatory conditions?

**Figure 5:**
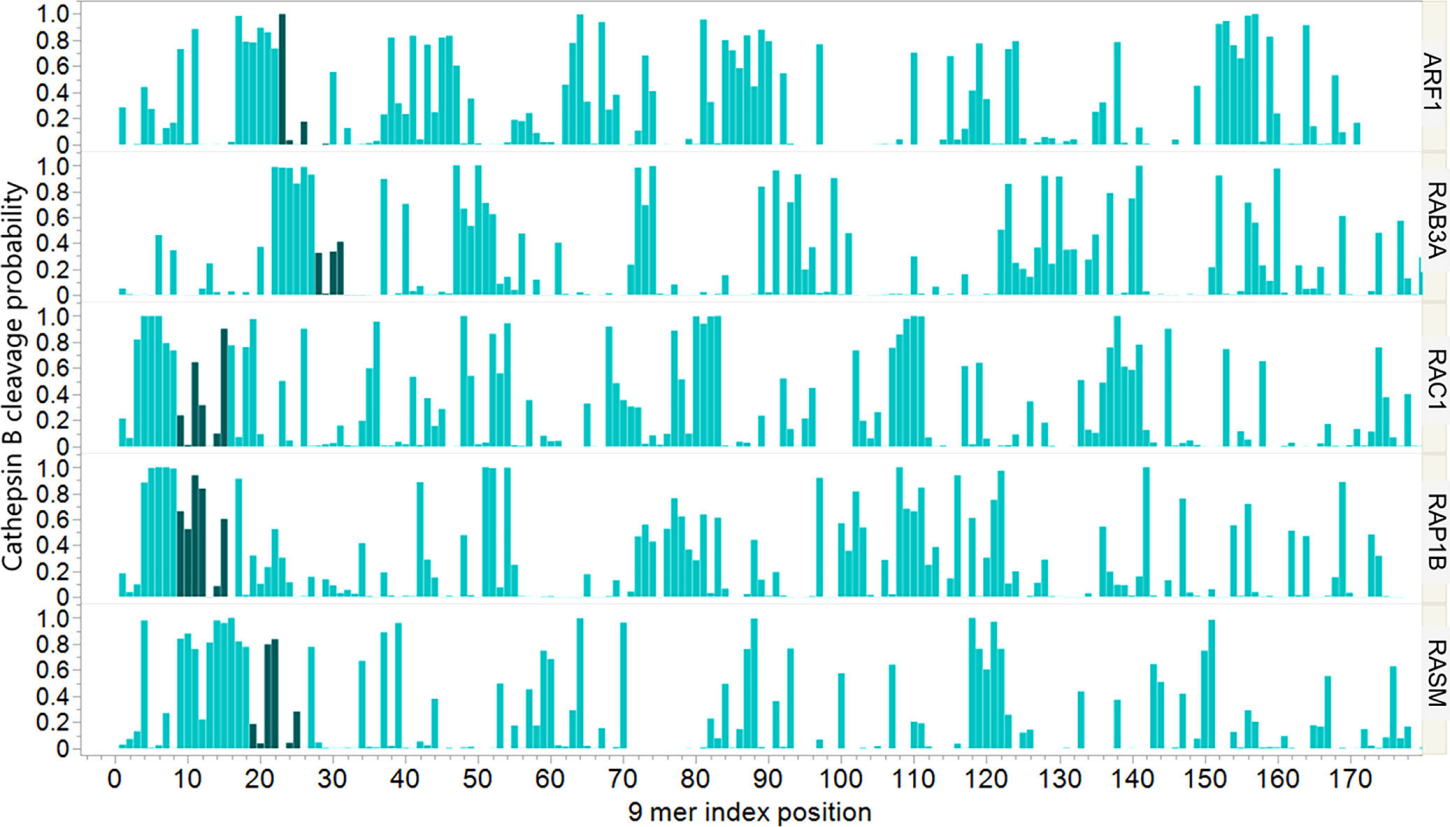
Predicted cathepsin B cleavage of a subset of other Ras family gene products. The X axis shows the index position of each sequential 9 mer peptide from N terminal of each protein shown on the right hand Y axis; proteins were truncated at 180 amino acids for comparison. The Y axis shows the predicted probability of cleavage of the peptide at the P1P1’ dimer comprising amino acids 4 and 5 of that 9 mer by cathepsin (B) The 9 mer peptides which comprise the GKS or GKT of a GXXXXGKS/T motif are shaded. The patterns show that peptides comprising the GXXXXGKS/T motif presented in a TCR exposed position have a high probability of cleavage.

The GXXXXGKS/T motif is characteristic of many small GTPases in addition to the KRas, NRas, and HRas oncogenes (14). Similar patterns of predicted cathepsin B cleavage exist in other GTPases (representative examples in **Figure 5**). This opens a broader speculative question of the relationship of inflammation to the control of other pathways in which the GTPases play a key role.

## 11 Conclusion

Bioinformatics provides a platform for conceptualizing, and testing, hypotheses involving multivariate causality. We have laid out a hypothesis that KRas, and related Ras, escape immune control because the critical neoepitopes are cleaved during inflammation and become immunologically invisible. We also raise questions about the molecular structural consequences to KRas of P loop cleavage, and the role of wild type KRas in tumorigenesis. We believe our observations, and the collective literature, support the hypothesis and form the basis for experimental testing.

## Data Availability

The original contributions presented in the study are included in the article/**Supplementary Material**. Further inquiries can be directed to the corresponding author.
