# Supplementary material for: The role of inflammation in the immune evasion of KRas
Source: Front Immunol. 2026 May 7;17:1831303. doi: 10.3389/fimmu.2026.1831303 (PMC13189885; doi:10.3389/fimmu.2026.1831303)
Supplement: Supplementary file 1 [file DataSheet1.pdf]

Supplementary Figures

Supplemental Figure 1: Predicted cleavage of KRas and different KRas mutants by cathepsin B at pH 6.

Neural network ensembles were developed using as a training set the data derived from mass spectrometry protein cleavage fragments of human cell culture homogenates that had been digested with cathepsin B (1, 2). A process of contrastive training was used where a cleavage site octamer with a scissile bond dipeptide from the training set was paired with another octamer with the same dipeptide from the same protein that was not digested. Neural network equation ensembles were developed predicting the cleavage probability between the P1P1' scissile bond using principal components of the physical properties of the cleavage site octamer amino acids in a bootstrap aggregation process (3). The predicted mean ± standard deviation of seven ensembles is plotted for each dipeptide in the protein molecule. Through application in mass spectrometry proteomics a threshold value of 0.8 indicated by the gray area in each figure has been determined to be a practical guide (3).

Supplemental Figure S1

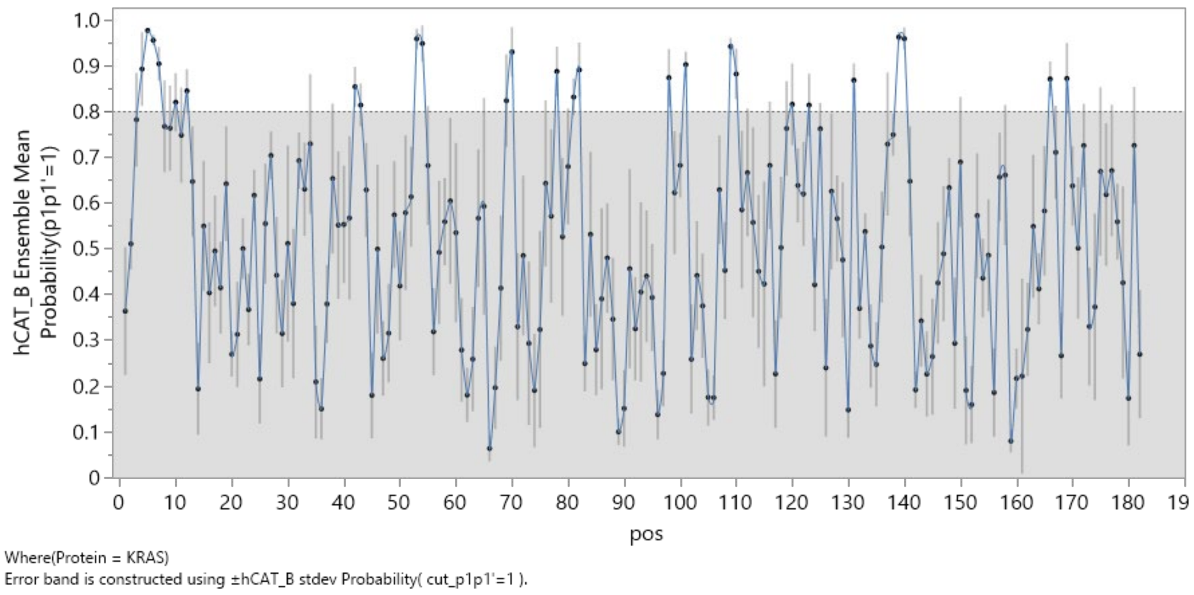

S1a: KRas wild type

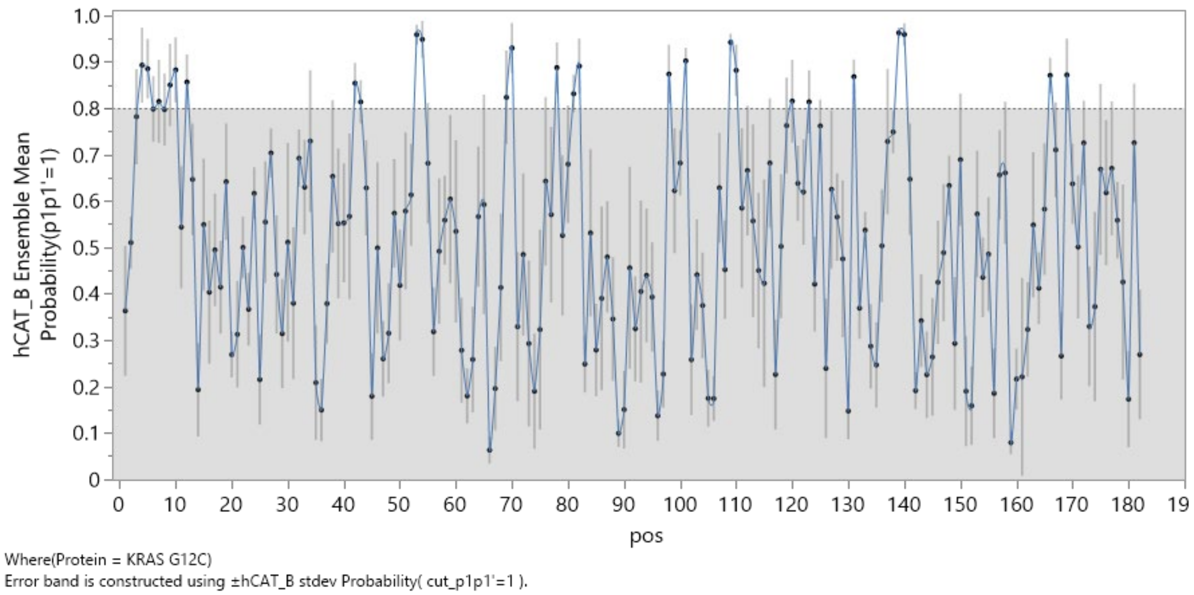

S1b: KRas G12C

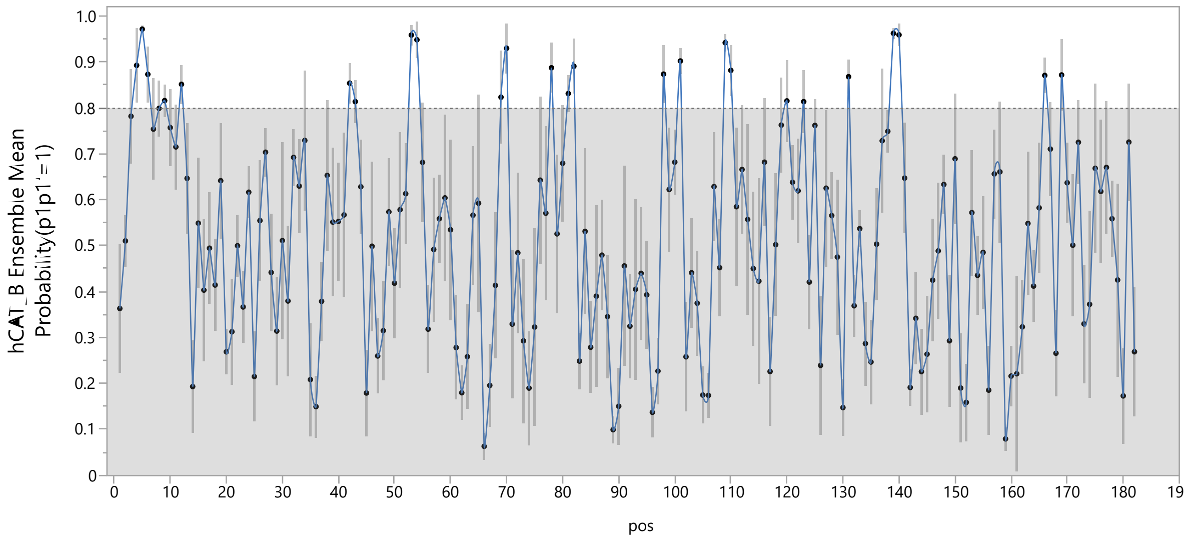

Where(Protein = KRAS G12D)

Error band is constructed using ±hCAT_B stdev Probability(cut_p1p1'=1).

S1c: KRas G12D

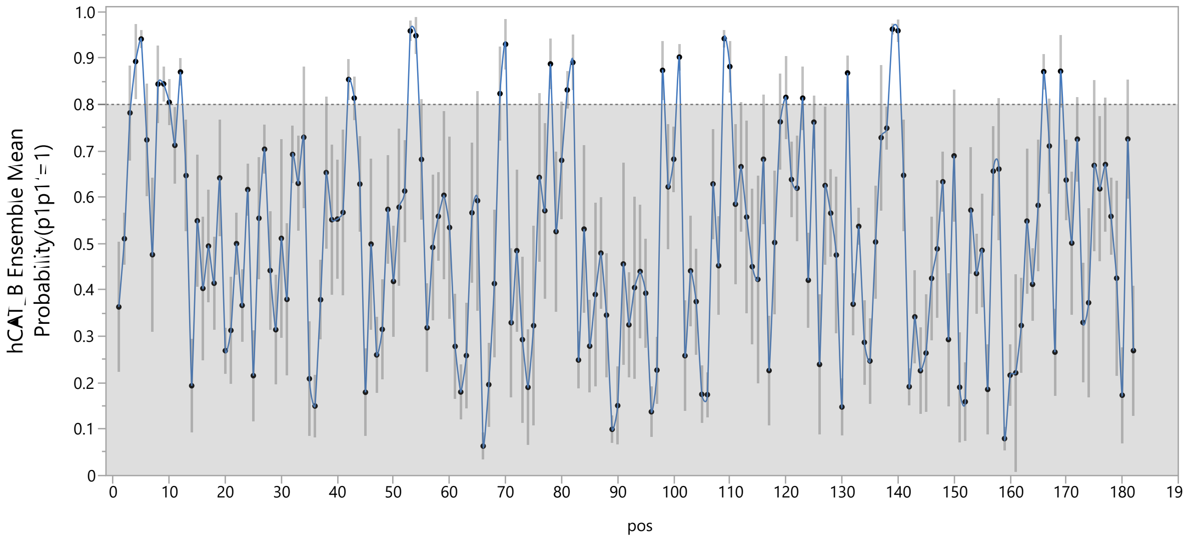

Where(Protein = KRAS G12R)

Error band is constructed using ±hCAT_B stdev Probability(cut_p1p1'=1).

S1d: KRas G12R

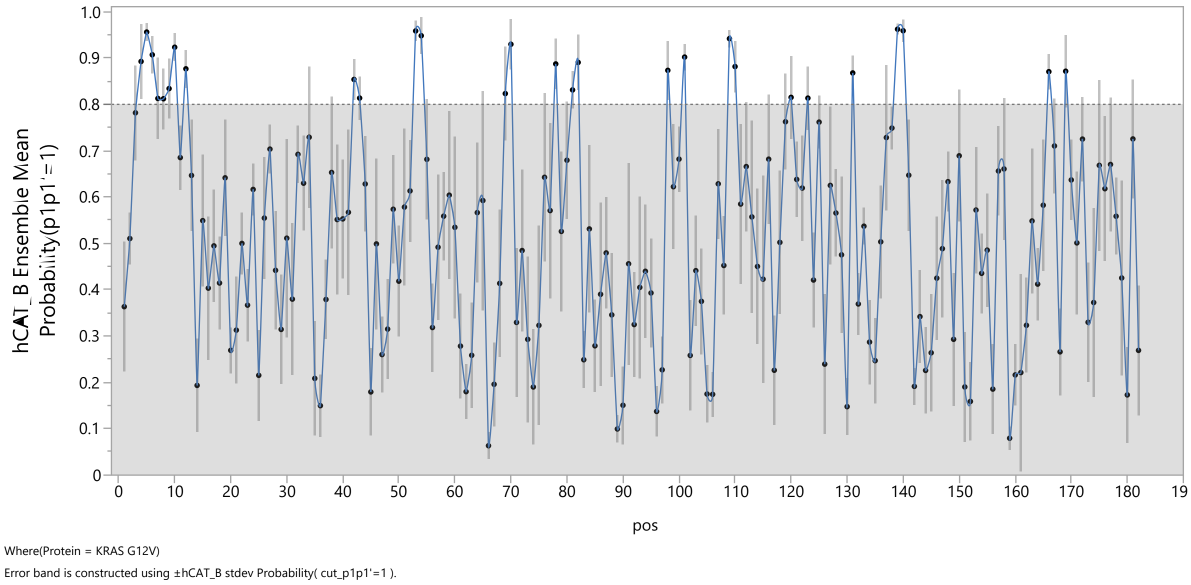

S1e: KRas G12V

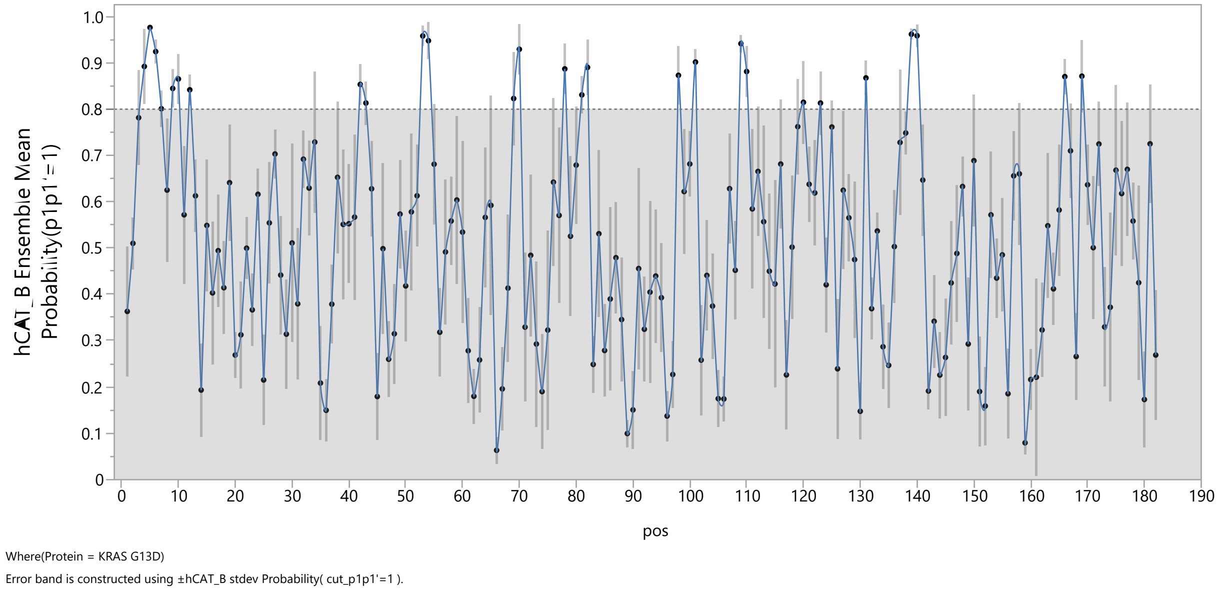

S1f: KRas G13D

Supplemental Figure S2: Predicted cathepsin B cleavage in TP53.

The X axis shows the index position of each sequential 9 mer peptide from N to C terminal of TP53. The Y axis shows the predicted probability of cleavage of the peptide at the P1P1' dimer comprising amino acids 4 and 5 of that 9 mer by cathepsin B.

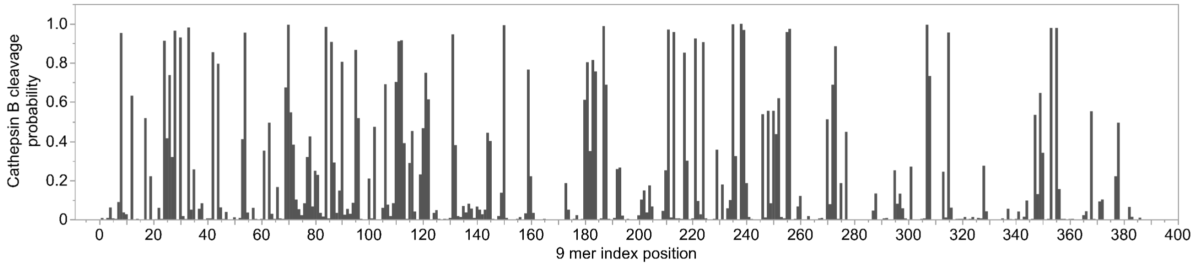

1. Tusar L, Loboda J, Impens F, Sosnowski P, Van Quickelberghe E, Vidmar R, et al. Proteomic data and structure analysis combined reveal interplay of structural rigidity and flexibility on selectivity of cysteine cathepsins. Commun Biol. 2023;6(1):450.

2. Biniossek ML, Nagler DK, Becker-Pauly C, Schilling O. Proteomic identification of protease cleavage sites characterizes prime and non-prime specificity of cysteine cathepsins B, L, and S. JProteomeRes. 2011;10(12):5363-73.

3. Hoglund RA, Torsetnes SB, Lossius A, Bogen B, Homan EJ, Bremel R, et al. Human Cysteine Cathepsins Degrade Immunoglobulin G In Vitro in a Predictable Manner. Int J Mol Sci. 2019;20(19).
